# Effects of the lateral amplitude and regularity of upper body fluctuation on step time variability evaluated using return map analysis

**DOI:** 10.1371/journal.pone.0180898

**Published:** 2017-07-10

**Authors:** Kazuhiro Chidori, Yuji Yamamoto

**Affiliations:** 1 Faculty of Nursing and Rehabilitation Department, Physical Therapy, Chubu Gakuin University: 2–1 Kirigaoka, Seki-shi, Gifu, Japan; 2 Graduate School of Education and Human Development, Nagoya University, Nagoya, Japan; 3 Research Center of Health, Physical Fitness and Sports, Nagoya University, Nagoya, Japan; Tokai University, JAPAN

## Abstract

The aim of this study was to evaluate the effects of the lateral amplitude and regularity of upper body fluctuation on step time variability. Return map analysis was used to clarify the relationship between step time variability and a history of falling. Eleven healthy, community-dwelling older adults and twelve younger adults participated in the study. All of the subjects walked 25 m at a comfortable speed. Trunk acceleration was measured using triaxial accelerometers attached to the third lumbar vertebrae (L3) and the seventh cervical vertebrae (C7). The normalized average magnitude of acceleration, the coefficient of determination ($R^2$) of the return map, and the step time variabilities, were calculated. Cluster analysis using the average fluctuation and the regularity of C7 fluctuation identified four walking patterns in the mediolateral (ML) direction. The participants with higher fluctuation and lower regularity showed significantly greater step time variability compared with the others. Additionally, elderly participants who had fallen in the past year had higher amplitude and a lower regularity of fluctuation during walking. In conclusion, by focusing on the time evolution of each step, it is possible to understand the cause of stride and/or step time variability that is associated with a risk of falls.

## Introduction

Healthcare for elderly populations has become a common problem worldwide because healthcare costs affect the economy in every country. Falls during gait are a major health issue in elderly people because they often lead to serious injuries such as hip fractures, hospitalization, and even death [1–3]. Within a 1-year period, falls occurred in over 30% of people older than 60 years living in the southern Tasmania [4]. Despite extensive preventative efforts, falls continue to be a major source of morbidity and mortality among the elderly [1,2].

Many studies concerning gait in elderly people have suggested that the coefficient of variation of gait speed and the one-step time are strongly associated with age-related changes and/or the risk of falls [5–8]. In particular, these studies suggest that stride time variability may be a better predictor of falls in the community-dwelling elderly than are other measures of motor function. Hausdorff et al. inserted foot pressure sensors in the shoes of community-dwelling elderly people and examined stride time variability as they walked on level ground for 6 min [6]. This 1-year prospective cohort study found that the stride time variability of fallers was significantly greater than that of non-fallers. However, the reason for this increase in temporal variability among fallers remains unknown.

Stride time variability is considered an index of the temporal variation in gait. Maki examined the spatiotemporal aspects of gait in elderly people and found that the standard deviations for stride width and stride variability in fallers were increased significantly compared with those of non-fallers [9]. Murray et al. also reported that increased step width caused by a decrease in lateral balance was a distinctive characteristic of gait in elderly people [10] and that decreased mediolateral posture control was strongly associated with the risk of falls [11,12]. Taken together, these findings suggest that, in the elderly, decreased mediolateral posture control results in not only temporal variability, such as in stride time, but also spatial variability, such as in step width, especially in fallers compared to younger people and non-fallers. Both temporal and spatial variability would increase the risk of falls in elderly people.

A possible cause of increased stride time variability is the movement amplitude of the center of gravity in relation to the variation of spatial factors. Control of head movement during gait stabilizes the optic flow, allowing more effective processing of vestibular system signals and, consequently, control of equilibrium. As Mazzà et al. described previously [13], in younger adults, acceleration of the upper body during walking was attenuated from the hips, through the pelvis and spinal column, and up to the head [13]. However, in the elderly, in general, and in those at high risk for falls, head acceleration was greater than pelvis acceleration, triggering instability when walking [13, 14]. A contrasting report found that the amplitude of lateral movement during gait fluctuated less in elderly people with a higher risk of falls because such subjects walked more carefully [15]. As described above, there is not necessarily a consistent relationship between the amplitude of lateral movement and stride time variability. Thus, regularity and/or fluctuation in trunk movement should be examined to clarify the cause of stride time variability in elderly people.

In this study, we defined fluctuation as the amplitude of trunk lateral movement during gait, and regularity as a stable temporal pattern of fluctuation regardless of amplitude. Time variability was defined as the coefficient of variation of the step time using a statistical method. For example, when the amplitude of trunk movement was increased by symmetrical breaking of lateral movement, and lateral fluctuation was regular, the step time variability on each side was smaller, regardless of stride variability. In contrast, when the symmetry of the trunk fluctuation was lost and lateral fluctuation was not regular, the step time variability was greater. This suggests the need to observe step-to-step symmetry and step time variability, not stride time variability [16–18]. That is, revealing regularity in the time evolution of trunk fluctuation could help to clarify the cause of step cycle fluctuations and fall risk.

The accelerometer has proven to be a reliable tool for measuring trunk fluctuation during gait for gait analysis [19]. For gait analysis, accelerometers have been attached to the vertex to examine the motion of the head, to the seventh cervical vertebra (C7) to measure the motion of the upper trunk [20,21], and to the third lumbar vertebra (L3) to measure the motion of the lower trunk [22,23]. These measurement locations reflect the movement of the body’s center of gravity, serving to capture the body movements at each site during gait. These measurements can be quantified for variability and fluctuation by analyzing the acceleration profiles obtained at each location for every gait cycle [24–26]. Recently, Terrier and Reynard showed that mediolateral (ML) direction measurements were more sensitive to deficits in balance control than were multidirectional measurements of local dynamic stability, a concept derived from chaos theory [27]. To quantify lateral regularity, autocorrelation coefficients (AC) have been used [23,28]. However, the value of AC depends on the amplitude and frequency of the acceleration profiles, although the value represents the overall gait cycle, not the time evolution during gait.

Return map analysis is used to reveal regularity hidden in a complex time series, based on a dynamic system perspective. The best-known return map is the Lorenz map. From a relatively short duration of continuous time series data, it determines distinctive variables, and it can evaluate the regularity of a system’s time evolution using the discretization method [29]. This method is not only applicable to different time series for a human individual [30,31]; it can also be applied to offensive and defensive behaviors between two people [32].

The purpose of this study was to classify the gait pattern into clusters in terms of the amplitude and regularity of trunk fluctuation and then to clarify the relationship between the cluster and gait variability, and further clarify the characteristics of elderly people who have experienced falls. The hypothesis was that those who have greater regularity and low lateral fluctuation in the upper body would show lower step variability. Conversely, those with large fluctuation and low regularity would show higher step variability. Additionally, elderly people who have low regularity and large fluctuation would be at an increased risk of falling.

## Materials and methods

### Participants

Eleven healthy, community-dwelling older adults (3 males, 8 females) and 12 younger adults (9 males, 3 females) participated in this study. The average age (SD) of the participants in the older adults group was 78.5 (5.0) years, and that of the younger adults group was 21.0 (0.6) years. Older adults were recruited through a local community center, and younger adults were recruited from a university between September and November 2014 in Gifu Prefecture, Japan. The demographic characteristics of the participants are shown in Table 1. All of the older adults were older than 65 years and were not certified by the government as needing support. Inclusion criteria were the ability to independently perform all activities of daily living, the ability to walk for 30 m without a break, and the ability to walk without any aid. Subjects with musculoskeletal and/or neurological diseases, any gait disorder, and/or any painful condition were excluded. In this study, according to the definition of Niino [33], falls were defined as "events that cause subjects to fall to the ground or other lower level against their will", which is merely a modification of the definition proposed by Gibson [34]. The older adults (only) were assessed using the Short Physical Performance Battery (SPPB) [35].

**Table 1:** Demographic characteristics of all subjects.

Characteristics	Younger adults (n = 12)	Older adults (n = 11)	*p* value
Age (year)	21.0 ± 0.6	78.5 ± 5.0	< 0.01
Gender, male/female (n)	9/3	3/8	
Height (m)	1.7 ± 0.1	1.6 ± 0.1	< 0.01
Weight (kg)	55.8 ± 8.3	57.4 ± 8.1	ns
Short physical performance battery	0.15	0.10	
(SPPB)	-	11.0 ± 1.5	
Fall history (n)	-	3	

Values shown are the means ± standard deviations. ns, non-significant

The Research Ethics Committee of Chubu Gakuin University approved the present study (Approval No. E14-0007). All subjects were informed in detail about the research protocol, and all gave written informed consent to participate in the study. The experimental procedures were conducted in accordance with the Declaration of Helsinki.

### Apparatus

Two wireless triaxial accelerometers (LP-WS1255A; range ± 5G, Logical Product Co., Fukuoka, Japan) were used to measure trunk movements during walking. To measure trunk acceleration, one accelerometer was attached over the L3 spinous process [22,23] (using an elastic belt), and an identical accelerometer was attached over the C7 spinous process (using a clavicle fixer band with surgical tape); movement was not restricted. A foot switch (a wireless-type foot sensor module; 4Assist, Inc., Tokyo, Japan) was used to record heel contact and toe-off events, and step duration was computed. Each footswitch contained four pressure sensors (FA-DL-250; 4assist Co., Tokyo, Japan) placed on both sides of the toes and the heel. All acceleration data and footswitch records were sampled at 200 Hz, and the two acceleration signals were synchronized with sole-pressure data. After analog-to-digital conversion, all of the signals were collected in a logger and immediately transferred to a laptop computer.

### Measurements

All subjects were instructed to walk on a smooth, horizontal 25-m walkway at a self-selected comfortable speed in bare feet. We analyzed the data collected over 20 consecutive steps in the middle 20 m of the walkway. Twenty consecutive steps were sufficient to apply return map analysis to examine the regularity of trunk fluctuations [30–32]. The duration taken to walk the central 10 m of the walkway was recorded using an electric stopwatch. The average gait speed was calculated by dividing this time by 10. A physical therapist was always present to ensure the safety of the older adults.

### Signal processing

ML trunk acceleration was analyzed in this study. Signal processing was performed using MATLAB Release 2010a (MathWorks, Natick, MA). All acceleration data were low-pass filtered using a dual-pass, zero-lag, fourth-order Butterworth filter with a cut-off frequency of 20 Hz. All heel contact and toe-off events were recorded by the foot switches. All analyses were performed using data from the middle 20 steps of steady walking in each trial. From these 20-step data, we first calculated the 20-step time for each step and thus the coefficient of variation for the 20 step times (step CV); this served as an index of gait variability. Next, we calculated the average magnitude of the acceleration (root mean square (RMS) of the acceleration); this revealed the average amplitudes of acceleration at L3 and C7 in ML direction, during a full walking trial. Since Menz et al. found that the acceleration of the body while walking increases with walking speed, they recommended using the normalized RMS (nRMS), *i*.*e*., the RMS standardized by dividing it by the square of walking speed, instead of RMS [26], we also calculated the normalized RMS (nRMS) values. To calculate the nRMS, we divided each RMS by the square of the walking speed.

### Return map analysis and cluster analysis

**Fig 1:**
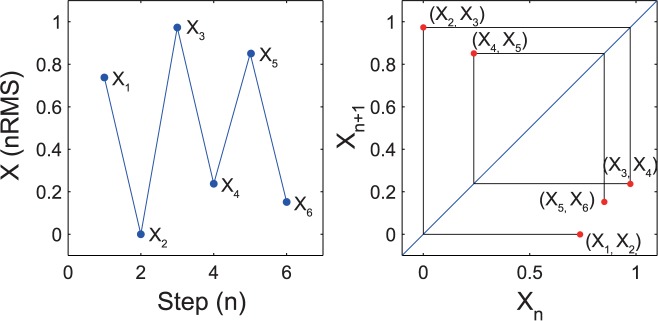
Return map analysis. Return map of the time series of the observed data (right panel), X_n_ versus X_n + 1_ using the amplitude of nRMS at each step corresponding to the series of points (blue circles) in the left panel.

Return maps were created to evaluate the time evolution of the step-by-step nRMS with standardized data. Each return map plotted the present nRMS (X_n_) against the next nRMS (X_n+1_), with *n* ranging from 1 to 19 (Fig 1). Such maps are commonly used to reveal regularities hidden in the time evolution of discrete dynamic systems [30,31]. The coefficient of determination (R^2^) was calculated to determine the goodness-of-fit of the simple regression line of the return map. This reflected the regularity of the time series of fluctuation in each step. A high R^2^ value was indicative of good regularity over the 20 steps. If a major difference was evident between the nRMSs of right and left steps, and if the nRMSs of all right and left steps are identical, then the R^2^ value is unity. As such, we used each map to explore regularity within the time evolution pattern of dynamic behavior during walking.

To classify the gait patterns using nRMS, which represents the magnitude of trunk fluctuation and regularity in the time series of trunk fluctuation, we used a cluster analysis. Cluster analysis is used widely to classify the gait patterns of healthy people and patients using EMG results, gait, posture, and kinematic variables [36–39]. Here, we applied it to trunk fluctuations during gait. We used the Ward method to perform a hierarchical cluster analysis with reference to the Euclidean distance [40]. Many studies have used a k-means cluster analysis to classify the distinct gait groups of patients with disorders such as cerebral palsy [38,41] and healthy participants. However, because we could not define the number of gait patterns of the elderly *a priori*, we chose a hierarchical clustering method instead of a non-hierarchical clustering method. We defined four clusters by combining the nRMS and regularity data using computed dendrograms. Regarding clinical validity, the dendrograms for C7 were drawn to identify the subjects who had experienced falls. Additionally, one-way analysis of variance was used to explore differences among the four clusters in terms of step CV, and the Bonferroni method was used to perform multiple comparisons.

### Statistical analyses

We explored the normality of distribution of all variables, including age, height, weight, gait speed, step CV, nRMS, and regularity, at L3 and C7. If normality was confirmed, we applied Student’s t-test; otherwise, the Mann–Whitney U-test was performed. Next, cluster analysis was used to reveal the relationships between the nRMS and regularity during walking. All statistical analyses were conducted using the ‘R’ software (ver. 3.2.2). The level of significance for all analyses was set at *p* < 0.05. The effect size was calculated in terms of Cohen’s *d*.

## Results

### Differences in gait variables between younger and older adults

Table 2 shows the differences in gait variables between the two groups. The walking speed was significantly faster in the younger adults (*p* = 0.03, d = 0.56), and the step CV was significantly greater in the older group (*p* = 3.49 × 10^−4^, d = 2.47). The normalized root mean square (nRMS) value indicating trunk fluctuation at L3 was 0.08 ± 0.01 in the younger group and 0.11 ± 0.03 in the older group; the older group’s value was significantly greater than that of the younger group (*p* = 0.03, d = 0.94). The nRMS value indicating trunk fluctuation at C7 in the younger group was 0.06 ± 0.02; in the older group, it was 0.11 ± 0.03. The value was significantly greater in the younger group (*p* = 4.54 × 10^−5^, d = 2.95). The time series regularity during walking was higher in the younger than in the older group only at L3 (*p* = 0.04, d = 0.86).

**Table 2:** Gait speed, step CV, nRMS and regularity at L3, C7 in the ML direction.

	Younger adults (n = 12)	Older adults (n = 11)	*p* value
Gait speed (m/s)	1.35±0.15	1.23±0.10	0.03
Step CV (%)	2.3±0.5	3.9±1.5	3.49×10^−4^
nRMS at L3	0.08±0.01	0.11±0.03	0.03
Regularity at L3 (*R*^2^)	0.11±0.12	0.35±0.33	0.04
nRMS at C7	0.06±0.02	0.11±0.03	4.54×10^−5^
Regularity at C7 (*R*^2^)	0.48±0.28	0.39±0.34	ns

Coefficient of determination (*R*^2^) to indicate the goodness-of-fit of the regression line in the return map. Values are means ± standard deviation. The data reflect the regularity of the time series of the average difference in stability between successive steps. CV, coefficient of variation; nRMS, normalized root mean square; L3, lower trunk; C7, upper trunk.

### Relationship between nRMS and regularity at L3 and C7

**Fig 2:**
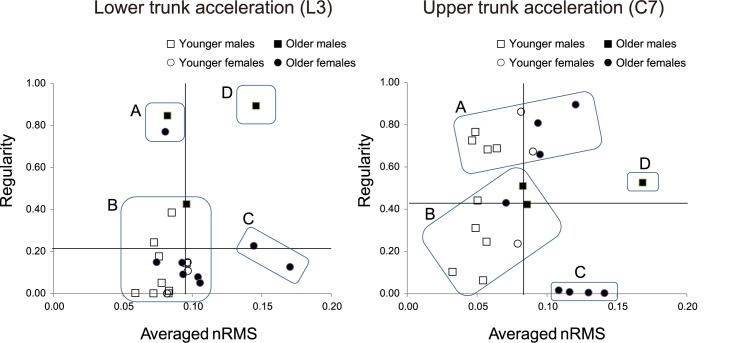
Relationships between the nRMS values of trunk acceleration and regularity in the ML direction. nRMS, normalized root mean square; ML, mediolateral. The solid line shows the average values. Filled circles show older adults, and open circles show younger adults.

Cluster analysis was performed using two variables, nRMS (representing fluctuations in the upper and lower trunk) and regularity (i.e., the regularity of the nRMS time series of each step). It revealed four clusters of lower (L3) and upper (C7) trunk acceleration. The statistical cluster validity based on internal validity was examined using the cophenetic correlation coefficient (CPCC) [42]. The CPCCs were 0.856 and 0.771 for L3 and C7, respectively. We used the upper tail method and the Jain-Dubes method to determine that there were four clusters (S1 File). Fig 2 shows the four clusters based on the relationship between nRMS and regularity. Cluster A was the combination of lower nRMS and higher regularity; cluster B was the combination of lower nRMS and lower regularity; cluster C was the combination of higher nRMS and lower regularity; and cluster D was the combination of higher nRMS and higher regularity. The clusters at L3, A, and C contained two older adults each; cluster D had only one older adults; and cluster B contained all of the younger adults and all of the remaining older adults. Thus, the cluster analysis based on L3 data did not identify the characteristics of subjects who walked unstably. In a comparison of the clusters at C7, the A and B clusters contained six younger adults and three older adults. Cluster C contained four older adults, and cluster D had one older adult. Although the three older adults who had experienced falls in the past year were in the B and C clusters at L3, they were all in the C cluster at C7. Thus, the clusters at C7 captured the characteristics of walking instability, unlike the clusters at L3. All subsequent analyses focused on the data for the clusters at C7 only.

**Fig 3:**
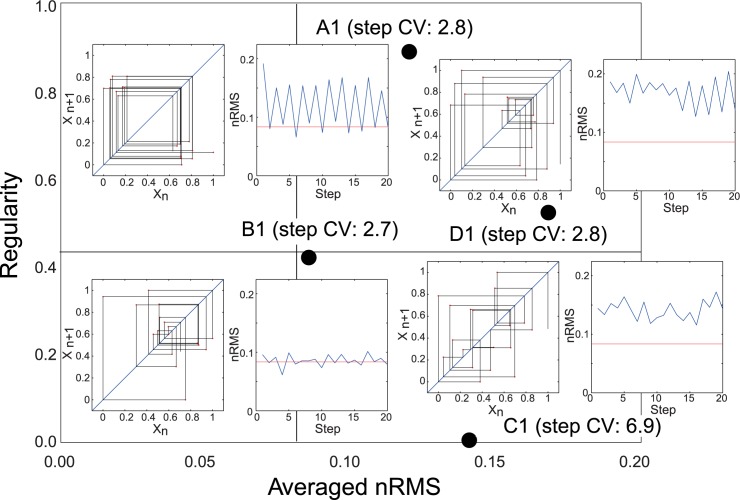
Examples of return map and time series for C7. Left side of each subfigure shows the return map. The *x*-axis is *Xn* times, and the *y*-axis is *Xn* + 1 times the amplitude of nRMS, which was standardized from 0 to 1 in each participant. The right side of each subfigure shows the time series of nRMS. Red lines show the mean of the averaged nRMS in pooled participants. The value in each example shows step CV in each participant.

Fig 3 shows typical examples of the return maps and time-series datasets for the older adults in each of the four clusters. The A cluster exhibited greater time series regularity, although the amplitude of trunk fluctuation was not reduced. The B cluster exhibited lower regularity in the time series, although the amplitude of trunk fluctuation was lower. The C cluster exhibited lower regularity and a higher amplitude of trunk fluctuation. Finally, the D cluster exhibited moderate regularity and a higher amplitude of trunk fluctuation. Thus, we identified four walking patterns in the mediolateral direction based on nRMS data and regularity during walking.

### Relationship between the clusters at C7 and step CV

**Fig 4:**
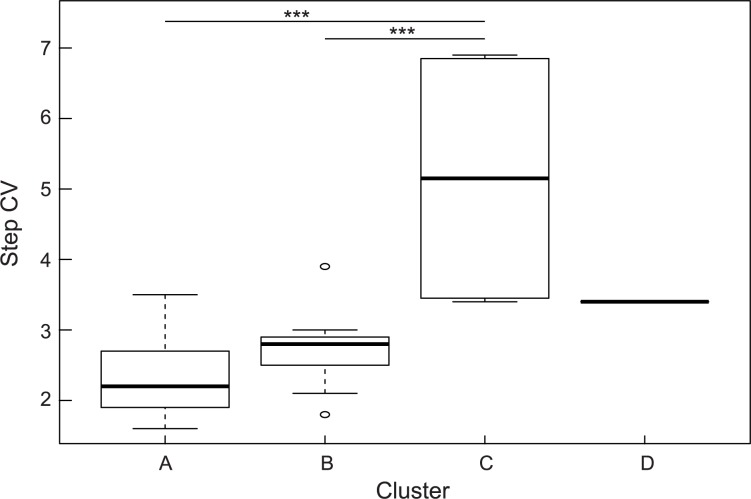
Boxplots of step CV for each cluster in the upper trunk (C7).

We tested the differences between clusters A, B and C by dividing the step CV on each side, but the difference between the clusters did not change. Therefore, statistical analysis was carried out over 20 steps on both sides (S1 Fig). To compare step CV values in the A, B, and C clusters at C7, we used a one-way analysis of variance and multiple comparison testing using the Bonferroni method. Because there was only one person in D cluster, it was excluded from statistical analysis. The results showed that the step CV of the C cluster was significantly higher than those of the A and B clusters (*p* = 2.40 × 10^−4^, d = 2.70 and *p* = 1.02 × 10^−3^, d = 2.36, respectively; Fig 4).

The C cluster was characterized by lower regularity and a greater amplitude of trunk fluctuation. This shows that lower regularity and higher trunk fluctuation were related to the step CV. Additionally, three of the four elderly subjects in the C cluster had fallen in the previous year. Thus, the data also indicated that a greater step CV increased the risk of falls. Next, we examined the relationship between falls and other indices, including non-normalized RMS; there was no significant difference between fallers and non-fallers in RMS or nRMS (S1 Table, S2 Fig). Taken together, these data suggest that lower regularity and greater fluctuation in the trunk during walking increase the risk of falls.

## Discussion

In human movements, variability plays an important role in adapting to environmental changes. Also, redundancy in the neuromuscular–skeletal system can accommodate many motor problems [43]. Dynamic stability in human movements, including walking, is underpinned by this variability and/or redundancy. Previous studies have focused on temporal variability in walking in relation to the risk of falls in elderly people. Certainly, too little and too much variability are not beneficial; however, some temporal variability during walking may contribute to adaptation to the diversity of the environment. The characteristics on which we should focus are spatial fluctuation in the amplitude of trunk lateral movements and temporal regularity relative to temporal variability. In this study, we focused on the amplitude and regularity of trunk fluctuations as factors related to step variability that increase the risk of falls in older adults. Additionally, we applied a return map analysis based on dynamic systems theory to trunk fluctuations to clarify the regularity of the time-related evolution. As a result, regularity of movement at L3, i.e., at the pelvis level, was lower in young than in older adults; however, regularity of movement at C7, i.e., at head level, was increased in both the young controls and older adults. This can be explained by the fact that older individuals attenuated fluctuation at the pelvis level to stabilize head movements [13].

Cluster analysis was applied to the amplitude and regularity of trunk fluctuations, which could be classified into four clusters: high amplitude and high regularity, high amplitude and low regularity, low amplitude and high regularity, and low amplitude and low regularity. The older adults showed larger trunk fluctuation amplitudes than the young controls did. This result is consistent with previous studies [13,14]. Regarding regularity at the pelvis level (L3), all of the young people showed scores <0.5 for regularity, as did 8 of the 11 older adults. This suggests that low regularity of lower trunk fluctuation was a common feature in both groups. However, in terms of regularity at the head level (C7), 6 of the 12 young controls and 6 of the 11 older adults showed scores <0.5 for regularity, and the others showed scores >0.5. This suggests that regularity at the head level varied regardless of age.

Young people with low trunk fluctuation amplitude and regularity showed low step variability. Conversely, the older adults with high trunk fluctuation amplitude and regularity showed low step variability. This could explain why previous studies did not find consistent results between the amplitude and variation in trunk fluctuation during the gait cycle [15]. That is, we could not evaluate which aspects of gait increase the risk of falls using only trunk fluctuation amplitude. However, when we added the regularity of the time evolution of fluctuation at the head level to the amplitude of trunk fluctuation, we could identify low step variability in the elderly. Additionally, the older adults with high trunk fluctuation amplitude and low fluctuation regularity had experienced a fall. Thus, the features of gait associated with a high risk of falls in older adults were not only high gait cycle fluctuation and high trunk fluctuation amplitude but also low regularity in the time evolution of the gait pattern.

These results partially supported our hypothesis that those who have highly regular lateral fluctuations in the upper body and low fluctuation amplitude would show low step variability, whereas those with large fluctuations and low regularity would show higher step variability. Contrary to our hypothesis, the older adults who had low trunk fluctuation regularity and amplitude showed low step variability. Thus, low trunk fluctuation would seem to decrease gait cycle variability. In addition, even if the older adults showed high trunk fluctuation amplitude, regularity of fluctuation would decrease gait cycle variability. This finding suggests that the combination of low upper trunk fluctuation amplitude and regularity would increase gait cycle variability and the risk of falls. In fact, we found that older adults who showed both low regularity of trunk fluctuation and large trunk fluctuations experienced falls.

In the present study, the regularity of the time evolution of trunk fluctuation and the amplitude of trunk fluctuation was examined from the viewpoint of dynamic systems theory. As a result, we clarified the relationship between trunk fluctuation and gait time variability and trunk fluctuation and the risk of falls. By examining gait in elderly individuals, we developed a new perspective on the time evolution of gait pattern. In terms of application, if we could find a new intervention to enhance trunk fluctuation regularity, it might be possible to reduce the risk of falls in the elderly.

However, there are several limitations to this research. First, the number of subjects was small, the ratio of males to females was biased, and the number who had experienced falls was small. Second, the older adults were people whose physical function was relatively well maintained, but it is known that the lower physical function is, the higher the risk of falls becomes. Thus, in the future, in addition to increasing the number of research subjects, research should also investigate older adults with poorer physical function.

## Supporting information

S1 Fig Comparisons of step time variability (step CV) among four clusters at C7 on each side. (PDF)

S2 Fig Relationships of gait speed with RMS, nRMS, regularity, and step time variability (step CV). (PDF)

S1 File Cluster validity. (PDF)

S1 Table Comparison of gait speed, step CV, nRMS and regularity in faller and non-faller. (PDF)
