# Supplementary figures and images for: Effects of the lateral amplitude and regularity of upper body fluctuation on step time variability evaluated using return map analysis
Source: PLoS One. 2017 Jul 10;12(7):e0180898. doi: 10.1371/journal.pone.0180898 (PMC5507271; doi:10.1371/journal.pone.0180898)

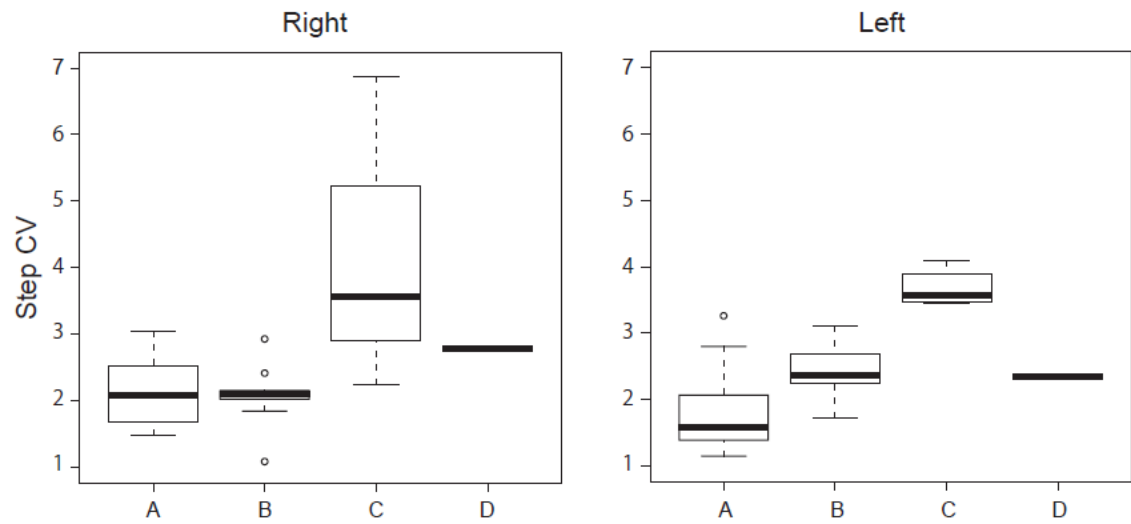

**Fig S1. Comparisons of step time variability (step CV) among four clusters at C7 on each side**

Supplement: S1 Fig — (PDF) [file pone.0180898.s001.pdf]

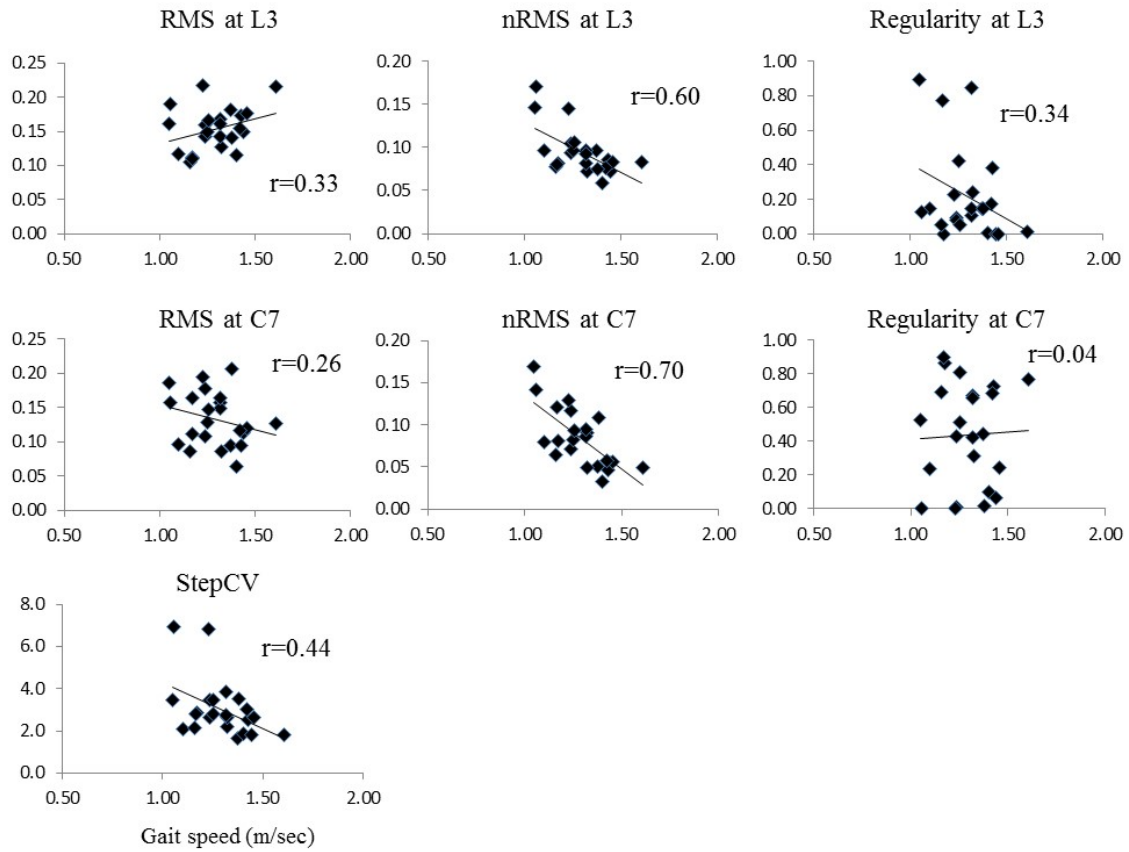

**Fig S2.**

Relationships of gait speed with RMS, nRMS, regularity, and step time variability (step CV) .

Supplement: S2 Fig — (PDF) [file pone.0180898.s002.pdf]
