# Supplementary material for: Effects of the lateral amplitude and regularity of upper body fluctuation on step time variability evaluated using return map analysis
Source: PLoS One. 2017 Jul 10;12(7):e0180898. doi: 10.1371/journal.pone.0180898 (PMC5507271; doi:10.1371/journal.pone.0180898)
Supplement: S1 File — (PDF) [file pone.0180898.s003.pdf]

Cluster validity

The number of clusters, k, needs to be specified explicitly for the k-mean algorithm. In most cases, however, k is not known beforehand. To find the optimal number of clusters, we used two cluster validity analyses: the Upper Tail method (Mojena, 1977) and the Jain-Dubes method (Davies & Bouldin, 1979; Jain & Dubes, 1988; Ngo, Pong, & Zhang, 2002).

Upper tail method

This method was proposed by Mojena (1977) and is an important cluster number decision procedure for hierarchical clustering using statistical stopping rules. These rules utilizes $N-1$ items in the distribution of the criterion (α) by calculating the mean and standard deviation of the sample. Only the distance is considered as a criterion; the values of the criterion can range from α_1, i.e., one cluster, to α_{N-1}. The stopping rule proceeds to define a 'significant' α as one that lies in the upper tail of the distribution satisfying

$$\alpha_j \leq \bar{\alpha} + k s_\alpha \tag{1}$$

where α_j represents the value of the criterion at stage j, k is the standard deviation, and $\bar{\alpha}$ and s_α are the mean and unbiased standard deviation of the α distribution, respectively. In Mojena (1977), k was varied from 2 to 4, which corresponded to datasets that included between 60 and 120 items. In this case, we chose $k = 2$.

Jain-Dubes method

Davies and Bouldin (1979) and Jain and Dubes (1988) and proposed a cluster separation measure that minimizes the intra-cluster distance, while maximizing the inter-cluster distance. The cluster separation measure $\rho(k)$ is defined as

$$\rho(k) = \frac{1}{k} \sum_{i=1}^{k} \max_{1 \leq n \leq k} \left\{ \frac{\eta_i + \eta_j}{\xi_{ij}} \right\} \tag{2}$$

where

$$\eta_j = \frac{1}{n_j} \sum_{i=1}^{n_j} D\left(\mathcal{F}_i^{(j)}, \mu_j\right) \tag{3}$$

$$\xi_{ij} = D(\mu_i, \mu_j) \tag{4}$$

μ_i is mean vector of cluster i, $\mathcal{F}_i^{(j)}$ is the ith vector in cluster j, and n_j is the number of vectors in cluster j. Thus, η_{ij} is the intra-cluster distance of cluster j, while ξ_{ij} is the inter-cluster distance of clusters i and j. k is set using Sturges' rule, that is, $2 \leq k \leq 1 + \log_2 n$. n is the number of data points. In this case, $k = \{2, 3, 4, 5, 6\}$, and the value that gives the lowest value of $\rho(k)$ is chosen.

Results

The results for the two methods are shown in Figure A and Tables A and B. The upper tail method revealed that a 'significant' α is $j = 3$. However, as shown in Table A, the value of α_j corresponding to $j = 4$ was equivalent to $j = 3$. The result of the Jain-Dubes method showed that the minimum value of $\rho(k)$ was 0.501, which corresponded to five clusters. The "best" choice of cluster number depended on the method.

Figure B shows the partitions corresponding to three to five clusters. Taken together, we chose four clusters as the "better" choice of cluster number. In other words, four clusters are relatively appropriate for our data.

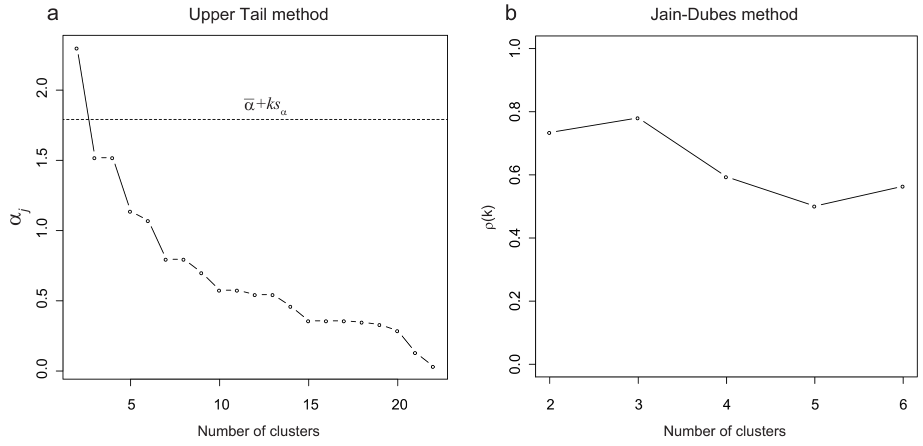

Figure A: **a**. Upper tail method. The dashed line shows $\bar{\alpha} + ks_\alpha$. **b**. The Jain-Dubes method

Table A: Values of criterion α_j at each stage

Number of clusters	2	3	4	5	6	7	8
α_j	2.299	1.519	1.519	1.138	1.071	0.796	0.796
Number of clusters	9	10	11	12	13	14	15
α_j	0.700	0.576	0.576	0.544	0.544	0.461	0.357
Number of clusters	16	17	18	19	20	21	22
α_j	0.357	0.357	0.348	0.330	0.287	0.131	0.033

Table B: Cluster separation measure $\rho(k)$ at each stage

Number of clusters	2	3	4	5	6
$\rho(k)$	0.734	0.780	0.594	0.501	0.564

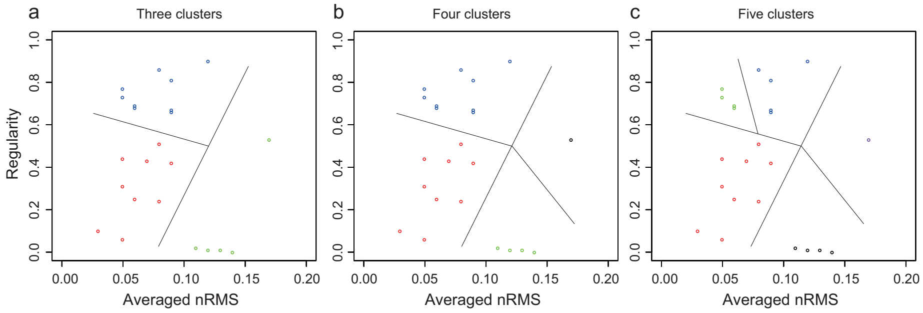

Figure B: Separation into three (**a**), four (**b**), and five (**c**).

Reference

Davies, D. L. & Bouldin, D. W. (1979). A cluster separation measure. *IEEE Transactions on Pattern Analysis and Machine Intelligence*, **PAMI-1**, 224–227.

Jain, A. K. & Dubes, R. C. (1988). *Algorithms for clustering data.* Englewood Cliffs, NJ: Prentice-Hall.

Mojena, R. (1977). Hierarchical grouping methods and stopping rules: an evaluation. *The Computer Journal*, **20**, 359–363.

Ngo, C.-W., Pong, T.-C., & Zhang, H.-J. (2002). On clustering and retrieval of video shots through temporal slices analysis. *IEEE Transactions on Multimedia*, **4**, 446–458.
