# Supplementary material for: Effects of the lateral amplitude and regularity of upper body fluctuation on step time variability evaluated using return map analysis
Source: PLoS One. 2017 Jul 10;12(7):e0180898. doi: 10.1371/journal.pone.0180898 (PMC5507271; doi:10.1371/journal.pone.0180898)
Supplement: S1 Table — (PDF) [file pone.0180898.s004.pdf]

Table S1.

Comparison of gait speed, step CV, nRMS and regularity in faller and non-faller.

		RMS		nRMS		nRegularity		Regularity		Speed	StepCV *
		L3	C7	L3	C7	L3	C7 *	L3	C7 *		
Faller	Mean	0.18	0.19	0.13	0.13	0.16	0.00	0.17	0.01	1.22	5.74
(n=3)	SD	0.04	0.03	0.05	0.02	0.03	0.00	0.05	0.01	0.16	1.93
Non-faller	Mean	0.15	0.15	0.10	0.10	0.42	0.52	0.41	0.53	1.23	3.15
(n=8)	SD	0.02	0.03	0.02	0.03	0.34	0.27	0.37	0.27	0.09	0.45

* $p<0.05$

RMS: non-normalized root mean square of the acceleration in ML direction.

nRMS: normalized root mean square of the acceleration in ML direction.

nRegularity: regularity was calculated using RMS.

Regularity: regularity was calculated using nRMS.

Speed: gait speed.

Step CV: coefficient of variation for the 20 steptimes.
